# Identification of Multiple Mechanical Properties of Laminates from a Single Compressive Test

**DOI:** 10.3390/ma15082950

**Published:** 2022-04-18

**Authors:** Bo Gao, Huai Yan, Boyi Wang, Qiang Yang, Songhe Meng, Yanyan Huo

**Affiliations:** National Key Laboratory of Science and Technology for National Defence on Advanced Composites in Special Environments, Harbin Institute of Technology, Harbin 150001, China; 18b918033@stu.hit.edu.cn (B.G.); 21b918095@stu.hit.edu.cn (H.Y.); 21b918021@stu.hit.edu.cn (B.W.); mengsh@hit.edu.cn (S.M.); 17b918040@stu.hit.edu.cn (Y.H.)

**Keywords:** property identification, laminate, delamination, sensitivity analysis, dynamic Bayesian network

## Abstract

In-plane elastic and interlaminar properties of composite laminates are commonly obtained through separate experiments. In this paper, a simultaneous identification method for both properties using a single experiment is proposed. The mechanical properties of laminates were treated as uncertainties and Bayesian inference was employed with measured strain-load curves in compression tests of laminates with embedded delamination. The strain–load curves were separated into two stages: the pre-delamination stage and the post-delamination stage. Sensitivity analysis was carried out to determine the critical properties at different stages, in order to alleviate the ill-posed problem in inference. Results showed that the in-plane Young’s modulus and shear modulus in elastic properties are dominant in the pre-delamination stage, and the interlaminar strength and type I fracture toughness in interlaminar properties are dominant in the post-delamination stage. Five times of property identification were carried out; the maximum coefficient of variation of identified properties was less than 1.11%, and the maximum error between the mean values of the identified properties and the ones from standard experiments was less than 5.44%. The proposed method can reduce time and cost in obtaining multiple mechanical properties of laminates.

## 1. Introduction

Carbon fiber reinforced composite has been widely used in the aerospace field, such as for the tail and fuselage of aircraft [1], due to its high specific strength, specific stiffness, and corrosion resistance [2,3]. Delamination is an important damage mechanism for this kind of material [4,5]. It is of great significance to obtain the mechanical properties of the laminate with delamination efficiently for evaluating the state of the laminate and predicting the remaining life [6,7,8]. The ability to obtain a single mechanical property, such as the elastic property and the interlaminar property, has formed a variety of standards. However, considering the cost of economy and time, how to obtain multiple properties through a single experiment has been of wide concern.

Molimard [9] identified multiple in-plain mechanical properties by using the tensile test of perforated thin plate, moiré technology and Levenberg-Marquardt (L-M) optimization method. Lecompte [10] carried out a biaxial loading test on glass fiber reinforced materials, obtained field strain information by an optical measurement method, and effectively identified multiple material performance properties based on a mixed numerical-experiment method. Lee [11] identified the elastic properties of flexibly supported rectangular laminated composite sandwich plates by using measured natural frequencies. Zhuo [12] proposed an inverse method to identify the mechanical properties of fiber metal laminates on the basis of the measured and calculated frequency response functions. Michopoulos [13,14,15,16] used the energy method to construct a general material constitutive model, and utilized the independently developed 6-DoF loading system and complex sample design to realize properties identification based on combined use of MATLAB and Ansys. The maximum difference between the obtained identification results and the standard test result was no more than 3.5%. Chen [17] proposed an inverse method for identification properties of variable stiffness composite laminates based on the approximate Bayesian computation, which can avoid the calculation of complex likelihood. Bouhala [18] proposed a method to identify the interface properties of unidirectional carbon/epoxy composite, based on the results of double cantilever beam and the corresponding a counterpart extended finite element method cohesive zone model. Su [19] developed an automatic identification of the interfacial cohesive properties between fiber-reinforced polymers and concrete based on a machine learning-based artificial neural network. Based on the full-field strain information obtained by digital image correlation and global sensitivity analysis method, Alfano [20] determined the main interface properties to be identified and the selection of the most suitable data area for identifying the properties. This provides guidance for the selection of appropriate observation data and experimental design.

Although much research has been done in identification of multiple properties of laminates, there is a lack of research on simultaneous identification of elastic properties and interlaminar properties in previous research. The reasons are as follows: (1) The key to identifying multiple properties through an experiment is that the experiment is sensitive to multiple properties. Therefore, how to design a single experiment sensitive to both elastic properties and interlaminar properties is a problem. (2) There are many elastic properties and interlaminate properties of laminates; identifying multiple properties at the same time can introduce the ill-posed problem due to the nature of the inverse problem. This means that the proper selection of the number of parameters to be identified at the same time is an important problem.

In this paper, a method of simultaneous identification of elastic properties and interlaminar properties is proposed, by using the characteristics of the test of the laminate with embedded delamination under compressive load. Whether the embedded delamination of the laminate extends under the compressive load is regarded as two different stages of parameter identification. Based on the results of sensitivity analysis (SA), the key elastic properties can be identified before the extension of the delamination under compressive load, and the key interlaminar properties can be identified after the extension of the delamination. In this way, two kinds of properties can be identified in one experiment, but the elastic and interlaminar properties are not identified at the same time, so as to alleviate the risk of an ill-posed problem of multiple properties identification.

## 2. Experimental Study

In this section, the response characteristics of the laminate with embedded delamination is illustrated by experiments, which can be regarded as the basis of the methodology proposed in Section 3.

### 2.1. Sample Preparation

**Figure 1:**
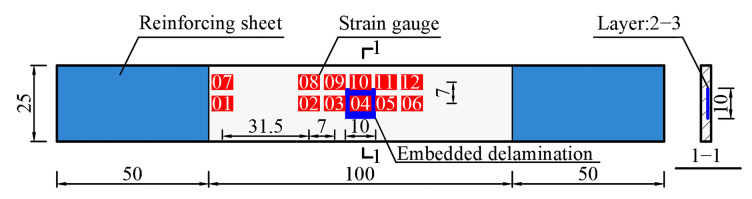
Parameter information of the laminate sample (Unit: mm).

The sample material of the laminate with delamination damage is carbon fiber reinforced epoxy composite. The laminate consists of 20 layers, each thickness is 0.104mm, and the layering sequence is [45/0//−45/0/45/90/−45/0/45/0/0/45/0/−45/90/45/0/−45/0/45] (//represents the position of the embedded delamination). The dimension properties of the laminate are shown in Figure 1. The delamination is a square with sides of 10 mm in the middle of the laminate. The embedded delamination was produced by placing polytetrafluoroethylene (PTFE) film (10 mm × 10 mm, 0.01 mm thickness) in the preset position before the material was cured and the material was cured by hot pressing method.

**Figure 2:**
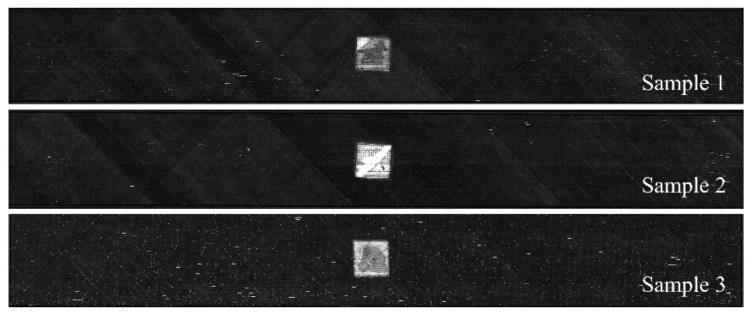
Ultrasonic scanning results of samples with delamination.

The ultrasonic scanning results of the delamination areas of three samples are shown in Figure 2. Considering the clamping requirements, 50 mm aluminum reinforcing sheets were pasted on both sides of the sample, and strain gauges were pasted along the loading direction at the designed sites, as shown in Figure 1. Strain gauges 1 and 7 were used to observe the strain characteristics at the end of the sample. Strain gauges 2–6 and 8–12 were used to observe the strain characteristics of the surface of the embedded delamination region and its adjacent areas under compressive loading.

### 2.2. Experiment Process

**Figure 3:**
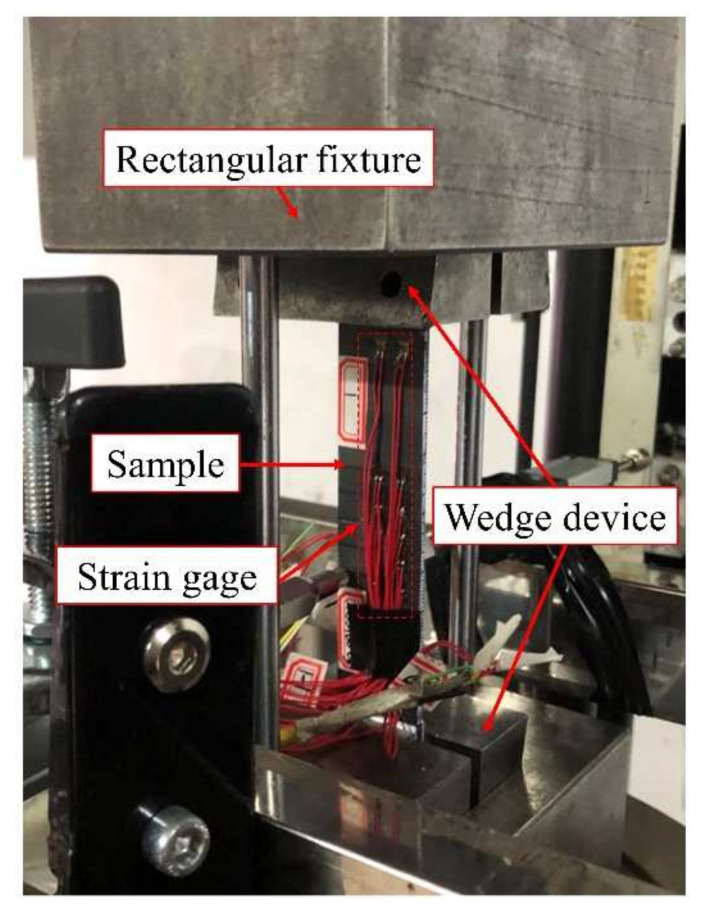
Test set-up for compressive testing.

**Figure 4:**
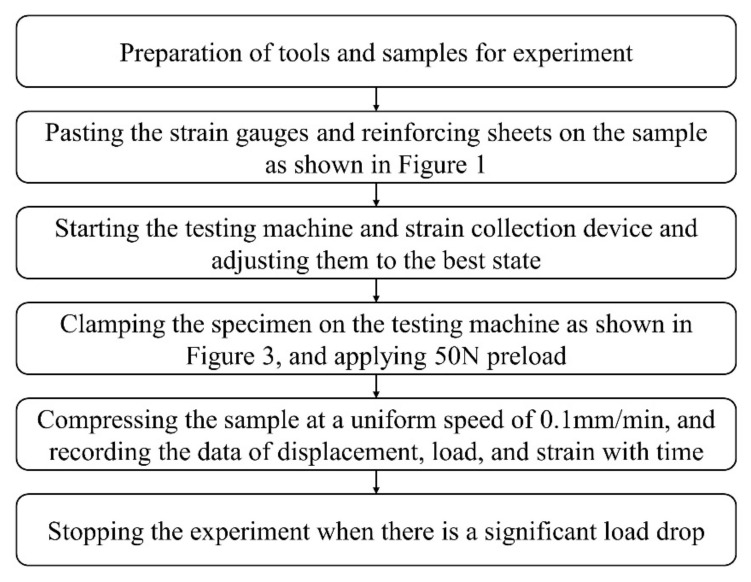
Experimental flowchart.

The experiment was carried out on a universal strength testing machine Zwick/Z100 (ZwickRoell, UIm, Germany). The experiment partly referred to ASTM D7137 [21] such as clamping strategy and loading rate, and the experimental device is shown in Figure 3. Both sides of the sample were clamped by a wedge device, and then the wedge device was assembled into a rectangular fixture with a wedge groove to facilitate the testing machine to apply the compressive load. The complete experimental process is shown in Figure 4. The whole test adopted displacement control loading, the loading rate was 0.1 mm/min, and the preload was 50 N. The strain information at different points of the sample was obtained by strain collection device, and the sampling frequency was 100 Hz.

### 2.3. Experiment Result

**Figure 5:**
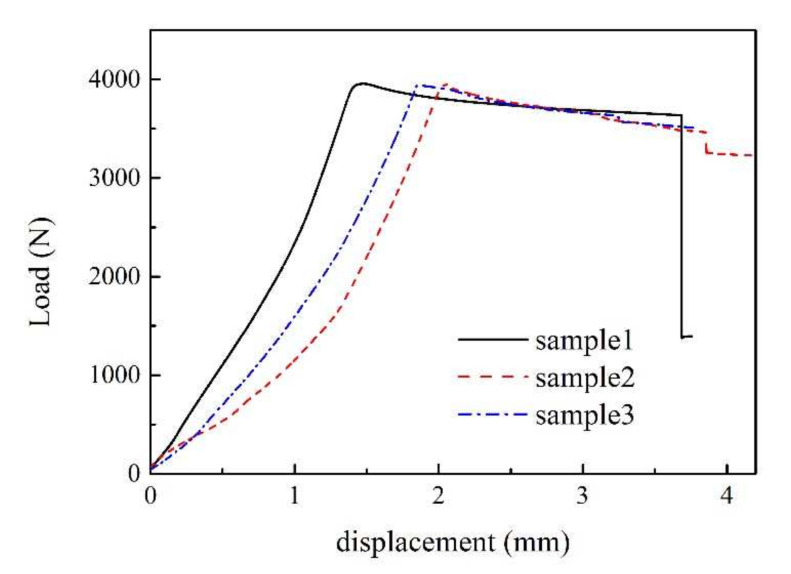
Load–displacement curves of three samples.

**Figure 6:**
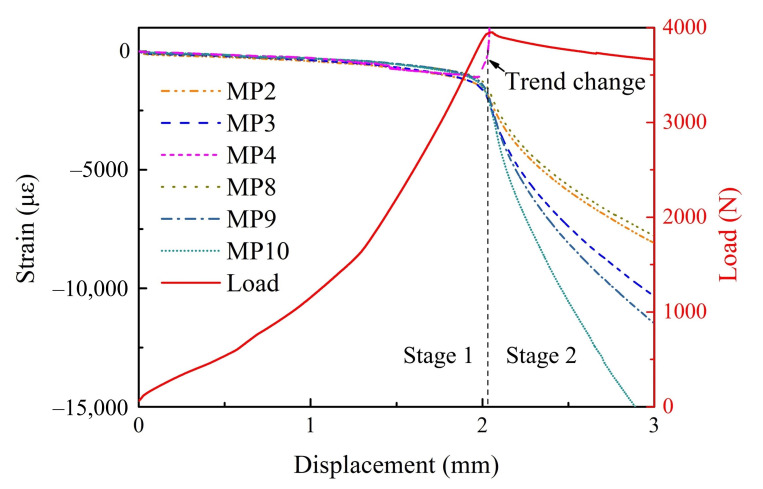
Characteristic comparison of the load–displacement curve and strain–displacement curves of multiple measuring points of sample 2.

The load–displacement curves of three experiments are shown in Figure 5. Taking sample 2 as an example, the strain–displacement curves at different measuring points (i.e., MPs in Figure 6) and the load–displacement curve are simultaneously given in Figure 6 for comparative analysis. It can be found that:

- Although the slope of the load–displacement curves of each sample was slightly deviate in the early stage due to the difference of fixture installation and the small variations in the production procedure of the samples, the peak load of all samples was close to 4000 N.
- The whole loading process can be divided into two stages: in stage 1, the load increased continuously, there was no local bucking, and the delamination did not extend; in stage 2, the load decreased gradually, local bucking began to occur, and the delamination began to extend in the direction perpendicular to the load. The information of local buckling can be proved by the strain characteristics of the MP4 pasted on the surface of the layered area as shown in Figure 1 (strain gage 04). When the load is near its peak, the strain of the MP4 changes rapidly from negative to positive, which represents a sudden change in the deformation characteristics of a region, from compression to tension, that is, outward buckling. Meanwhile, the slopes of strain–displacement curves of other MPs also changed greatly, representing the further intensification of the bending characteristics of the structure.

## 3. Methodology

### 3.1. Property Identification Method

#### 3.1.1. Framework

**Figure 7:**
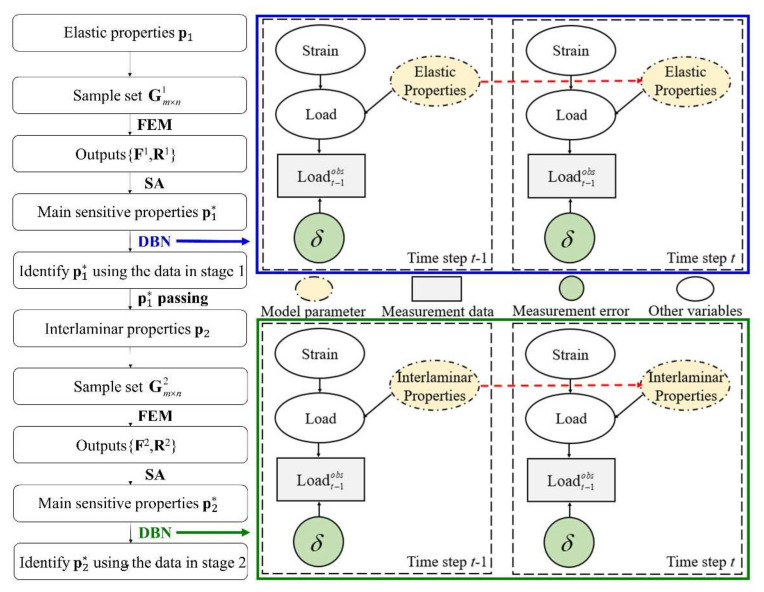
The framework of property identification method.

Considering the structural state of the laminate with embedded delamination under compressive load can be divided into two stages. In stage 1, there is no delamination expansion; the carrying capacity of the laminate is mainly reflected in the elastic properties of the material. In stage 2, due to the delamination expansion caused by local buckling, the carrying capacity is reflected in the elastic properties and interlaminar properties of the material. Therefore, based on such characteristics, a segmented method for identifying mechanical properties of the laminate is proposed. The elastic properties of the material are identified in stage 1, the calibrated properties are passed to stage 2, and the interlaminar properties of the material are identified in stage 2. The framework of parameter identification method is shown in Figure 7. The steps are as follows:

1. Based on the prior information of the material properties, the initial distributions of *m* elastic properties $p_{1}$ of the material are given (elastic properties including Young’s modulus, shear modulus, Poisson’s ratio, etc.). Then, *n* samples are randomly sampled according to the distribution characteristics of each property and randomly grouped as $G_{m\times n}^{1}$. Then, $G_{m\times n}^{1}$ is put into the finite element model (FEM) and the load response $F^{1}$ and the strain response $R^{1}$ of the measured points in Figure 1 are obtained.
2. The load sensitivity varying with the strain of each property is calculated on the basis of method of SA according to the sample ($G_{m\times n}^{1}$) and the outputs ($F^{1}$ and $R^{1}$). Thus, the main sensitive properties $p_{1}^{*}$ are determined.
3. Based on the dynamic Bayesian network (DBN), $p_{1}^{*}$ is identified using the time varying load–strain data of stage 1 in the experiment.
4. The identified properties are regarded as the real mechanical properties of the material. Step 1 is repeated among the *l* interlaminar properties $p_{2}$ (interlaminar properties including fracture toughness, interlaminar strength, etc.), forming the group $G_{l\times n}^{2}$ and outputs (load response $F^{2}$ and the strain response $R^{2}$).
5. Determine the main sensitivity properties $p_{2}^{*}$ of interlaminar properties according to step 2. Then, $p_{2}^{*}$ is identified using the time varying load–strain data of stage 2 in the experiment based on the DBN.

#### 3.1.2. Sensitivity Analysis Method

In this paper, the global sensitivity analysis (GSA) is adopted because it can consider the influence of the distribution interval of inputs on the outputs. There are many GSA methods [22,23,24,25,26,27]; the random balance design Fourier amplitude sensitivity test (RBD-FAST) [25] has been adopted in this paper. The FAST method is briefly introduced as follows:

Let Y=f(x) be a model output with *m* random inputs ($x=[x_{1},x_{2},\ldots ,x_{m}]$), and assume that all the inputs can form a unit hypercube. Each input is represented by a given search function ($G_{i}(\cdot )$):

(1) $$x_{i}\left(s\right)=G_{i}\left(\sin\omega_{i}s\right)\begin{array}{cc} & \forall i=1,2,\ldots ,m \end{array},$$

where *s* is a scalar variable varying over the range -π<s<π, $\omega_{i}$ is a specific frequency for each input variable, and $G_{i}(\cdot )$ is the common search function as follows [27]:

(2) $$x_{i}={\bar{x}}_{i}e^{{\bar{v}}_{i}\sin\omega_{i}s}\begin{array}{cc} & i=1,2,\ldots ,m \end{array},$$

(3) $$x_{i}={\bar{x}}_{i}\left(1+{\bar{v}}_{i}\sin\omega_{i}s\right)\begin{array}{cc} & i=1,2,\ldots ,m \end{array},$$

(4) $$x_{i}=\frac{1}{2}+\frac{1}{\pi }\arcsin\left(\sin\omega_{i}s\right)\begin{array}{cc} & i=1,2,\ldots ,m \end{array}.$$

The outputs (*Y*) can be expanded into a Fourier series:

(5) $$Y=f(x)=f_{s}\left(s\right)=A_{0}+\sum_{k=1}^{+\infty }\left[A_{k}\cos\left(ks\right)+B_{k}\sin\left(ks\right)\right].$$

For any positive integer ($\omega_{i}$), the period *T* is 2*π*. The corresponding Fourier coefficients are defined as follows:

(6) $$\begin{array}{l} A_{0}=\frac{1}{2\pi }\int_{-\pi }^{\pi }f_{s}\left(s\right)ds,\\ A_{k}=\frac{1}{\pi }\int_{-\pi }^{\pi }f_{s}\left(s\right)\cos\left(ks\right)ds,\\ B_{k}=\frac{1}{\pi }\int_{-\pi }^{\pi }f_{s}\left(s\right)\sin\left(ks\right)ds. \end{array}$$

The spectrum of the Fourier series is defined as follows:

(7) $$\Lambda_{k}=(A_{k}^{2}+B_{k}^{2})/2,k\in N^{*},$$

The first order of the variance-based global sensitivity of $x_{i}$ can be defined as:

(8) $$S_{i}=\frac{\sum_{p=1}^{+\infty }\Lambda_{p\omega_{i}}}{\sum_{k=1}^{+\infty }\Lambda_{k}},k,p\in Z,i=1,2,\ldots ,m.$$

In the FAST method, the $\omega_{i}$ needs to satisfy the linearly independent, which makes the computational cost of high-dimensional problems unbearable. To reduce the computational cost, the random balance design method is combined with the FAST [25]. In RBD-FAST, the $\omega_{i}$ takes the same value for each input variable. The method distinguishes the characteristics of the different inputs by controlling the ordering of the inputs without calculating a large number of samples, which can reduce the computing cost effectively. The process can be summarized as follows:

1. Determine $s=[s_{1},s_{2},\ldots ,\;s_{N}]$ and sort **s** randomly. The disordered sample of **x** is then obtained by random permutation of **s**, and the model outputs (*Y*) are calculated by a disordered sample of **x**.
2. The outputs (*Y*) are rearranged according to the original sequence of the sample of $x_{i}$. The Fourier transform is then performed according to Equation (5).
3. The first-order sensitivity index corresponding to $x_{i}$ can be obtained through Equations (6)–(8). Return to step 2 to determine the sensitivity of other inputs.

#### 3.1.3. Dynamic Bayesian Network

There are many classical methods for parameter identification, such as the iterative regularization method [28], the Tikhonov regularization method [29], and Bayesian method [30]. When the problem of parameter identification has the characteristics of large number parameters to be identified, model complex and time-dependent data, dynamic Bayesian networks (DBNs) are an excellent choice [31]. Therefore, a DBN is adopted in this paper for parameter identification.

A DBN is an extension of a Bayesian network (BN) in time domain. A BN is a directed acyclic graph model used for uncertainty inference. In a BN, random variables are represented vertices and their dependence are represented by directed edges. The quantitative characterization of dependence can be expressed by a probability density function or a definite function [32]. Compared to a BN, a DBN has additional lines connecting the same variable between two adjacent time points. This allows a DBN not only to integrate a BN content, but also to accumulate previous knowledge. There are many inference algorithms that can be used for a DBN, including multiple Kalman filters [33,34,35] and particle filters (PFs) [36]. Considering the no-linear characteristics of the model studied in this paper, PFs is selected as the inference algorithm of the DBN. The sequential importance resampling (SIR) algorithm, which is one of the PF algorithms, is employed in this paper.

**Figure 8:**
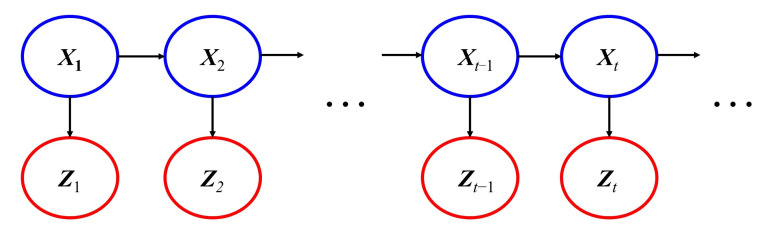
A simple example of a DBN.

The DBN depicted in Figure 8 is used to briefly introduce the SIR algorithm. As shown in Figure 7, the state variable ***X*** evolves over time according to the state function:

(9) $$X_{t}=f(X_{t-1},\varepsilon_{t-1}),$$

where ***ε*** is noise terms in the state function. The measurement variable ***Z*** is calculated by the measurement function:

(10) $$Z_{t}=g(X_{t},\eta_{t}),$$

where ***η*** is noise terms in the measurement function. The SIR algorithm is as follows [37]:

1. The initial particles ${\left\{x_{0}^{i}\right\}}_{i=1}^{N}$ are generated according to the prior probability density function (PDF).
2. Loop the following steps at *t* = 1, 2, …, :
  (1) Sampling from the proposal PDF: generating particles ${\left\{{\tilde{x}}_{t}^{i}\right\}}_{i=1}^{N}$ and calculating the corresponding weights ${\tilde{\omega }}_{t}^{i}$ according to $x_{t}^{i}\sim p(x_{t}^{i}|x_{t-1}^{i})$ and $\omega_{t}^{i}\propto \omega_{t-1}^{i}p(Z_{t}|x_{t}^{i})$.
  (2) Resampling and estimating: Particles set ${\left\{{\tilde{x}}_{t}^{i},{\tilde{\omega }}_{t}^{i}\right\}}_{i=1}^{N}$ is resampled to ${\left\{x_{t}^{i},1/N\right\}}_{i=1}^{N}$. Then, estimate the state at time *t*: ${\hat{x}}_{t}=\sum_{i=1}^{N}{\tilde{x}}_{t}^{i}{\tilde{\omega }}_{i}^{i}$.

### 3.2. Finite Element Simulations

**Figure 9:**
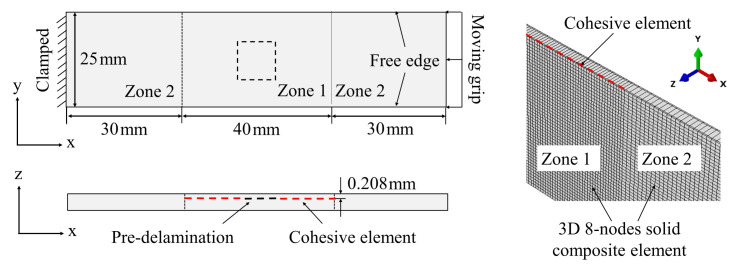
Finite element modeling, boundary and loading conditions for uniaxial compressive load.

The finite element model of the laminate has been established based on the commercial software ABAQUS (Dassault Systemes SIMULIA, Paris, France). The modeling procedure are shown in Figure 9. The 8-nodes 3D solid composite elements are used to model the laminate (C3D8RC3), which can provide accurate interlaminar stress and transverse shear effect [38]. To predict the delamination evolution, the interface between two sub-laminates is modeled by 8-nodes 3D cohesive elements (COH3D8). The volume thickness of the cohesive element is zero, and the bilinear traction-separation law has been employed herein (refer to [39]). In Figure 9, the left side of the laminate is completely fixed ($U_{x,y,z}^{}=0$), and the compressive displacement is applied to the right side ($U_{y,z}^{}=0$). The laminate is then divided into two zones artificially to save the computing cost. In zone 1, where sub-laminate local buckling occurs and delamination may propagate, the cohesive element has been positioned in the area as shown in Figure 9. Surface-to-surface contact element has been placed in the delamination zone to avoid overlaps between elements. The minimum mesh size is 0.5 mm. In zone 2, only global buckling occurs and the minimum mesh size is 0.75 mm. The quadratic stress failure criterion (QUADs) has been used as the delamination initiation criterion to calculate the delamination evolution under mixed stress state [38], which is expressed as:

(11) $${\left(\frac{T_{n}}{T}\right)}^{2}+{\left(\frac{T_{s}}{S}\right)}^{2}+{\left(\frac{T_{t}}{S}\right)}^{2}=1,$$

where *T*, *S* are the maximum tractions in the normal, and shear directions, respectively. $T_{n}$, $T_{s}$, $T_{t}$ are the traction components in the normal, first, and second shear directions. 

When the damage occurs, the damage evolution continues in the cohesive zone. An energy-based evolution model was adopted in this paper. Considering the mixed-mode behavior, the power-law criterion was used [38], which is expressed as:

(12) $${\left(\frac{G_{I}}{G_{\mathrm{IC}}}\right)}^{\alpha }+{\left(\frac{G_{\mathrm{II}}}{G_{\mathrm{IIC}}}\right)}^{\alpha }+{\left(\frac{G_{\mathrm{III}}}{G_{\mathrm{IIIC}}}\right)}^{\alpha }=1,$$

where $G_{\mathrm{IC}}$, $G_{\mathrm{IIC}}$, $G_{\mathrm{IIIC}}$ are the critical energy release rates (fracture toughness) for mode I, II, III, respectively. $G_{I}$, $G_{\mathrm{II}}$, $G_{\mathrm{III}}$ are the corresponding release rate values in analysis and *α* is a coefficient.

The nonlinear solution of the problems presented here is performed using standard ABAQUS procedures. The grid independence verification has been verified by 1.4 times number of grids, and the results of load–displacement curves are consistent. Note that the initial size and position of the embedded delamination are fixed in the simulation of this paper. The mechanical properties discussed in Section 3.1.1, including elastic properties and interlaminate properties, are the variable inputs of the model, which affect the response characteristics of the structure under displacement load. Other parameters remain unchanged during the simulation.

### 3.3. Verification Example

The verification example is based on the experiment in Section 2, and the process of the verification example is as follows:

1. The initial distribution intervals of 12 material properties of the laminate are shown in Table 1. A total of 1000 samples are randomly selected for each property, and the samples of each property are randomly combined to form a 1000 sample set $G_{12\times 1000}^{1}$. Then, bring $G_{12\times 1000}^{1}$ into the finite element model in Section 3.2 to obtain the strain response of the concerned measuring points under different loads.
2. The sensitivities of properties of the laminate at MP9 (just for illustration, other measuring points can also be used) under different strains in stage 1 are analyzed to obtain the key property $p_{1}^{*}$. Then, $p_{1}^{*}$ is identified based on the DBN along the loading process as shown in Figure 6. The observation points in DBN are shown in Table 2 and the observation error is set as 1% of the observation data (Observation data is obtained from MP9 in stage 1).
3. Similar to step 1, resample 1000 samples of interface properties to form the set $G_{6\times 1000}^{2}$. The sensitivities of properties of the laminate under different strains in stage 2 are analyzed to obtain the key property $p_{2}^{*}$. Then, $p_{2}^{*}$ is brought into the finite element model to calculate the strain response of MP9. At this time, the input elastic properties of the model are the properties just identified. $p_{2}^{*}$ is identified along the loading process in DBN as shown in Figure 7. The observation points are shown in Table 2 and the observation error is set as 1% of the observation value (Observation data is obtained from MP9 in stage 2).

There are some notes as follows: (1) All the mechanical properties are used in the sampling in step 1 for SA in the example, the purpose of which is to further prove that the interface parameters have no effect in the stage 1. (2) All the samples are simulated by finite element method. The total time for simulating a case is about 1 h, where stage 1 takes 40% of the time and stage 2 takes 60% of the time (CPU: Inter(R) Xeon(R) CPU E5-2695, 2.10 GHz, 16 cores are used in the calculation).

## 4. Results and Discussion

**Figure 10:**
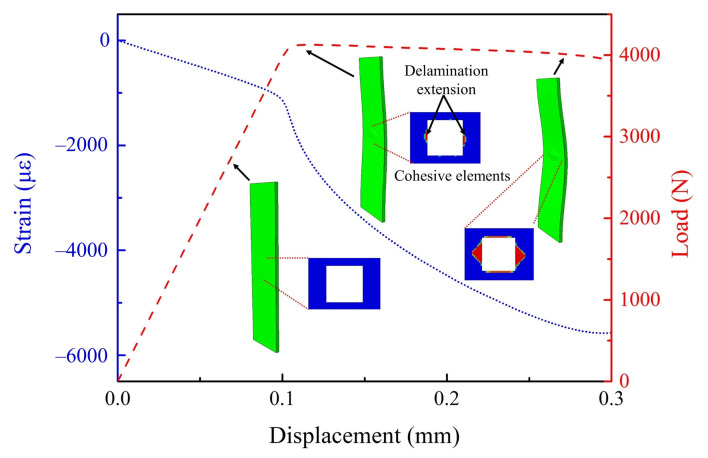
Finite element simulation results of the laminate with embedded delamination.

First, a sample ($E_{1}$ = 165.56 GPa, $G_{12}$ = 4.17 GPa, $G_{\mathrm{IC}}$ = 240.94 N/m, *T* = 10.09 MPa, *S* = 53.85 MPa) in step 1 of Section 3.3 was randomly selected to show the results of its finite element calculation, as shown in Figure 10. The characteristics of the load–displacement curve and the strain–displacement curve at MP9 are completely consistent with the corresponding curves of the experiment in Figure 6. In the simulation, it can be clearly seen that near the inflection point of the load–displacement curve, the delamination expansion caused by local buckling begins to occur, which further verifies the conclusion given by the experiment. As the load gradually increases, the delamination gradually extends along the direction perpendicular to the load, which is consistent with the conclusions of [38,39].

**Figure 11:**
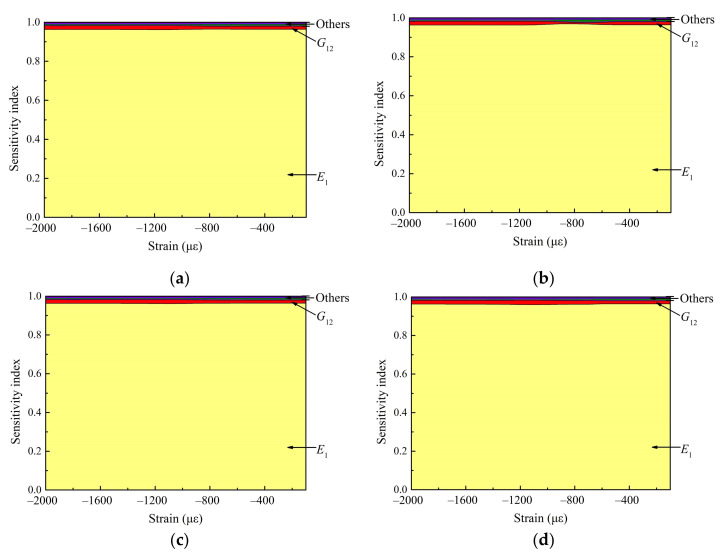
The SA results of each property to load under different strains in stage 1. (**a**) SA results at MP2; (**b**) SA results at MP3; (**c**) SA results at MP8; (**d**) SA results at MP9.

Then, the property sensitivity in stage 1 is shown in Figure 11. It can be seen that the sensitivity characteristics of properties do not change significantly at different MPs. Therefore, the calculation example only uses the data of MP9 to illustrate the method. In stage 1, the dominant properties are $E_{1}$ and $G_{12}$, and $E_{1}$ is absolutely dominant in the whole mode. This is mainly because the laminate does not occur delamination extension in stage 1, the deformation of the structure along the loading direction is more obvious under compression, and the direction of strain extracted is the same as the loading direction. Therefore, the modulus along the loading direction $E_{1}$ and the in-plane shear modulus related to the loading direction $G_{12}$ have important influence on the load.

According to the results of SA, only properties $E_{1}$ and $G_{12}$ need to be identified in stage 1. Then, based on the DBN, data of five observation nodes have been used to identify $E_{1}$ and $G_{12}$. In order to ensure the robustness of the identification results, a total of five identifications were carried out and their mean values were compared with those obtained by standard tests as shown in Table 3. It can be seen that the coefficient of variation of the two properties is less than 0.73%, and the maximum error with the properties obtained from the standard tests is no more than 1.57%, which proves the robustness and accuracy of the identification method.

**Figure 12:**
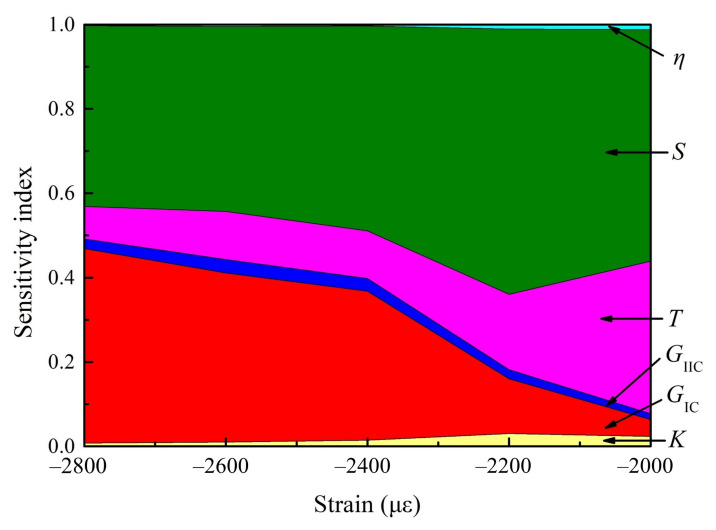
The SA results of each property to load under different strains at MP9 in stage 2.

**Figure 13:**
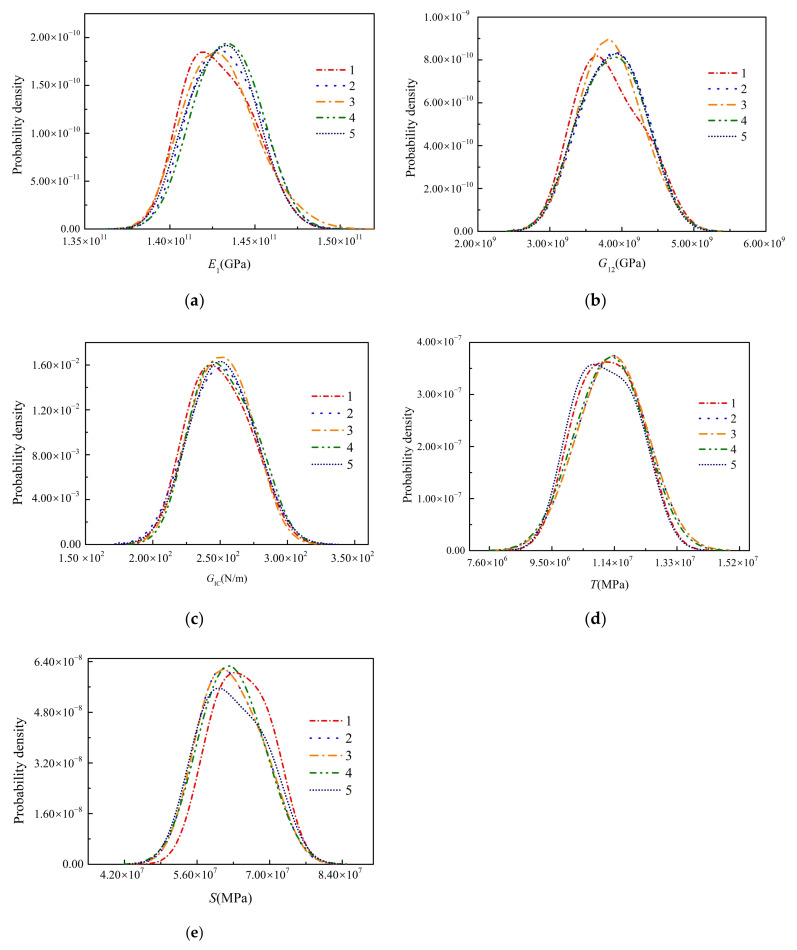
The posterior distribution of the properties obtained by five different identifications. (**a**) $E_{1}$; (**b**) $G_{12}$; (**c**) $G_{\mathrm{IC}}$; (**d**) *T*; (**e**) *S*.

The property sensitivity in stage 2 is shown in Figure 12. At this time, only the sensitivity of interlaminar properties is considered, and the elastic properties obtained by stage 1 are taken as the true values of the model. In stage 2, the dominant properties are $G_{\mathrm{IC}}$, *T*, and *S*. With the increase of the strain (i.e., the gradual extension of the delamination), the sensitivity of $G_{\mathrm{IC}}$ gradually increases while $G_{\mathrm{IIC}}$ does not. This shows that the delamination characteristics of the laminate under compressive load are mainly related to the mode of $G_{\mathrm{IC}}$. To our surprise, the sensitivity of penalty stiffness *K* and Power-law parameter *α* can be ignored. The identification results of the three interlaminar properties are shown in Table 3 and Table 4. The coefficient of variation of the three properties is less than 1.11%, and the maximum error with the properties obtained from the standard tests is no more than 5.44%. The posterior distribution of the five identified properties is shown in Figure 13. It can be found that the posterior distribution characteristics of the properties of five times identification are similar, which further proves the sampling 1000 particles can meet the robustness requirements of the method.

In order to show that the segmented parameter identification method proposed in this paper can improve the accuracy of parameter identification, the identification results of parameters were compared with the results of Levenberg-Marquardt (L-M) method, which is a classical method in iterative regularization [9]. When using the L-M method, five main parameters are identified at the same time. The comparison results are shown in Table 4. It can be found that the accuracy of parameter identification using the proposed method is higher than that using the L-M method, which identifies five parameters at the same time. The maximum error of the identification results of the two method is 5.44% to 8.61%.

**Figure 14:**
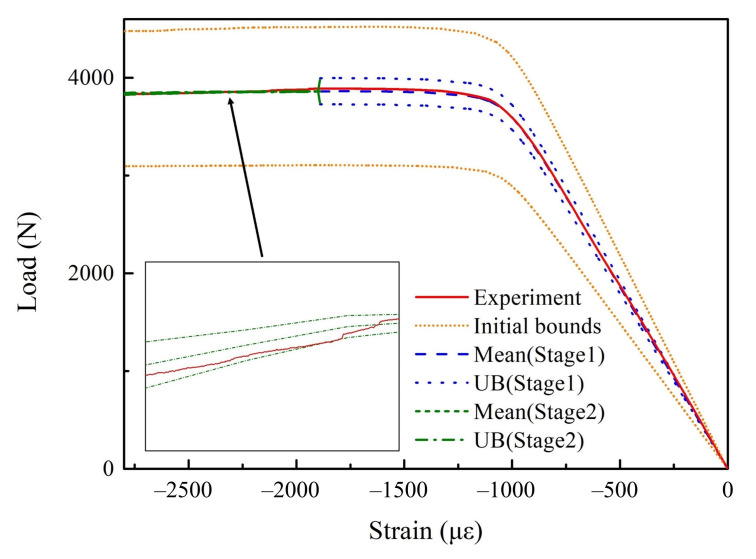
Comparison of load–strain curves between results of the 1st identification and experiment.

Compare the load–strain curve of the first identification result (i.e., $E_{1}$ = 142.85 GPa) with the experiment as shown in Figure 14. In Figure 14, Experiment means the experimental curve; Initial bounds represent the upper and lower bounds of load–strain curves for the initial distribution properties; Mean (Stage 1) and Mean (Stage 2) represent the mean value of the identification results of stage 1 and stage 2, respectively; UB (Stage 1) and UB (Stage 2) represent the uncertainty bounds of the identification results in stage 1 and stage 2, which is calculated by mean ± 2 × standard deviation and mean ± 4 × standard deviation, respectively.

It can be seen that the load–strain curves obtained by property identification are in good agreement with the experimental data. The uncertainty interval of load–strain curve formed by identification can effectively envelop the experiment data. After determining the key elastic properties ($E_{1}$,$G_{12}$) in stage 1, the whole uncertainty interval is significantly reduced. This means the elastic properties play a crucial role in the load–strain curve, and further explains the significance of obtaining the elastic properties of the material before identifying the interlaminar properties.

**Figure 15:**
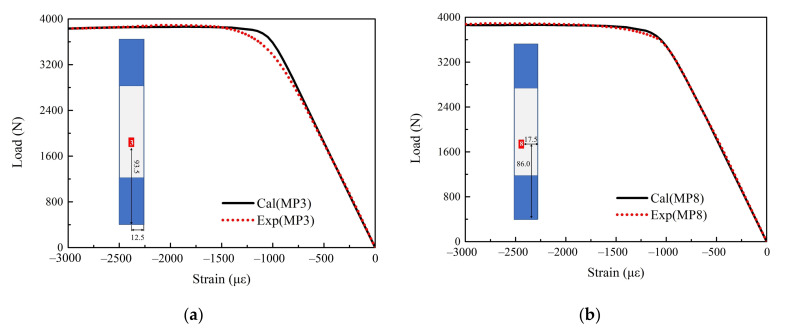
Comparison between identification results and experimental load–strain curves. (**a**) Comparison between the identification result and the experimental load–strain curve at MP3; (**b**) comparison between the identification result and the experimental load–strain curve at MP8.

Furthermore, the mean value of the identification results is brought into the finite element model to obtain the load–strain curves at other measuring points, and the curves are compared with corresponding experimental data as shown in Figure 15. In Figure 15, Cal(MP3) and Cal(MP8) represent the results of FEM at MP3 and MP8, respectively; Real(MP3) and Real(MP8) represent the experiment data at MP3 and MP8, respectively. It can be found that the identified properties can effectively characterize the strain-load curves of other measuring points, but there is a certain deviation when the strain reaches around 1250με (inflection point of the strain-load curve). This is mainly due to the following two reason: (1) the discreteness of material properties at different positions; (2) the finite element model is too ideal to fully reflect all the real state when the structural state sudden changes.

## 5. Conclusions

In this paper, a multiple mechanical properties identification method is developed based on the features that the response of defective laminate under compressive load has two stages. In stage 1, there is no extension of the delamination. The results of SA show that only two elastic properties $E_{1}$, $G_{12}$ need to be identified, and $E_{1}$ has a dominant position in the whole mode. In stage 2, the delamination begins to extend, and the results of SA show that the key interlaminar properties to be identified are fracture toughness $G_{\mathrm{IC}}$, interlaminar strength *T*, *S*. The sensitivity of $G_{\mathrm{IC}}$ increases with the increase of delamination expansion. The penalty stiffness *K* and power-law parameter *α* have little effect in the whole stage 2.

In order to show the robustness and accuracy of the property identification method, five attempts at property identification were carried out and the results were compared with the results of the standard tests. The results show that the maximum coefficient of variation of the five identified properties was less than 1.11%, and the maximum error of the mean of identification results compared with the standard tests was less than 5.44%. Furthermore, in order to prove that the segmented parameter identification method proposed in this paper can improve the accuracy of parameter identification, the identification results of parameters were compared with the results of L-M method, which identifies five parameters at the same time. The results show that the accuracy of parameter identification using the proposed method was better (maximum identification error 5.44% vs 8.61%).

After determining the key elastic properties $E_{1}$, $G_{12}$ in stage 1, the whole uncertainty interval of strain-load curve tracking is significantly reduced in stage 2. This means the elastic properties play a crucial role in both two stages. Identifying the interlaminar properties based on the identified elastic properties can improve the identification accuracy.

In future work, specific regions could be selected for property identification according to sensitivity characteristics based on the measurement technology of digital image correlation, so as to improve the sensitivity of multiple properties and the identification accuracy at the same time.

## Figures and Tables

**Table 1:** Laminate material properties.

Mechanical Magnitudes		Initial Distribution
Longitudinal Young’s modulus (GPa)	$E_{1}$	(140.00, 160.00)
Transverse Young’s modulus (GPa)	$E_{2}$	(8.90, 10.88)
Shear modulus (GPa)	$G_{12}=G_{13}$	(3.02, 4.57)
	$G_{23}$	(2.56, 3.84)
Poisson’s ratio	$v_{12}=v_{13}$	(0.25,0.38)
	$v_{23}$	(0.24,0.36)
Penalty stiffness (GPa)	*K*	(2.40, 3.60)
Interlaminar tensile strength (MPa)	*T*	(10.00,12.50)
Interlaminar shear strength (MPa)	*S*	(55.00, 72.00)
Fracture toughness (N/m)	$G_{\mathrm{IC}}$	(225.00, 285.00)
	$G_{\mathrm{IIC}}$	(465.76, 698.64)
Power-law parameter	*α*	(1.60, 2.40)

**Table 2:** Observation site information in property identification (MP9).

Observation Points	Stage 1 (Strain(με), Load (N))	Stage 2 (Strain(με), Load (N))
Point 1	(−1000.00, 3631.09)	(−2300.00, 3855.90)
Point 2	(−1220.44, 3833.85)	(−2400.00, 3849.69)
Point 3	(−1380.80, 3870.32)	(−2500.00, 3843.72)
Point 4	(−1540.55, 3882.84)	(−2600.00, 3840.20)
Point 5	(−1700.99, 3887.61)	(−2700.00, 3835.44)

**Table 3:** The results of property identification conducted five times.

Parameters	$E_{1}\left(\mathrm{GPa}\right)$	$G_{12}\left(\mathrm{GPa}\right)$	$G_{IC}\left(N/m\right)$	T(MPa)	S(MPa)
1	142.85	3.91	249.62	11.20	64.42
2	143.37	3.85	251.71	11.13	62.81
3	143.02	3.89	250.83	11.33	62.59
4	143.08	3.87	250.95	11.34	62.61
5	143.24	3.83	252.63	11.26	62.70
Mean	143.11	3.87	251.15	11.25	63.03
Coefficient of variation (%)	0.13	0.73	0.40	0.71	1.11

**Table 4:** The identification results of mechanical properties under different methods.

Parameters	The Results of Standard Test	The Results of Proposed Method/Error	The Results of L-M Method/Error
$E_{1}(\mathrm{GPa})$	141.00	143.11/1.50%	144.74/2.65%
$G_{12}(\mathrm{GPa})$	3.81	3.87/1.57%	3.48/8.61%
$G_{IC}(N/m)$	241.60	251.15/3.95%	224.90/6.91%
T(MPa)	10.67	11.25/5.44%	9.99/6.31%
S(MPa)	60.03	63.03/5.00%	54.98/8.41%

## Data Availability

The data presented in this study are available from the corresponding authors upon reasonable request.

## References

[1] Zimmermann R., Klein H., Kling A. (2006). Buckling and postbuckling of stringer stiffened fibre composite curved panels—Test and computations. Compos. Struct..

[2] Zniker H., Ouaki B., Bouzakraoui S., EbnTouhami M., Mezouara H. (2022). Energy absorption and damage characterization of GFRP laminated and PVC-foam sandwich composites under repeated impacts with reduced energies and quasi-static indentation. Case Stud. Constr. Mater..

[3] Zhang Y.Z., Huang K., Sun R.Q., Liao F., Guo L.C., Zhang L. (2022). Effect of embedded delamination on the compression performance of carbon fiber reinforced composites. Compos. Struct..

[4] Rozylo P. (2022). Comparison of failure for thin-walled composite columns. Materials.

[5] Jensen S.M., Martos M.J., Lindgaard E., Bak B.L.V. (2019). Inverse parameter identification of n-segmented multilinear cohesive laws using parametric finite element modeling. Compos. Struct..

[6] Panasiuk K., Dudzik K. (2022). Determining the stages of deformation and destruction of composite materials in a static tensile test by acoustic emission. Materials.

[7] Hanif A., Diao S., Pei H.F., Li Z.J., Sun G.X. (2019). Green Lightweight Laminated Cementitious Composite (LCC) for Wind Energy Harvesting—A novel application of LCCs. Case Stud. Constr. Mat..

[8] Zanelato E.B., Alexandre J., Azevedo A.R.G.D., Marvila M.T. (2019). Evaluation of roughcast on the adhesion mechanisms of mortars on ceramic substrates. Mater. Struct..

[9] Molimard J., Riche R.L., Vautrin A., Lee J.R. (2005). Identification of the four orthotropic plate stiffnesses using a single open-hole tensile test. Exp. Mech..

[10] Lecompte D., Smits A. (2007). Mixed numerical-experimental technique for orthotropic parameter identification using biaxial tensile tests on cruciform specimens. Int. J. Solids Struct..

[11] Lee C.R., Sun S.J., Kam T.Y. (2015). System properties of flexibly supported laminated composite sandwich plates. AIAA J..

[12] Zhuo X., Hui L., Wang W.Y., Liu Y.N., Wen B.C. (2019). Inverse identification of mechanical properties of fiber metal laminates. Proc. Inst. Mech. Eng. Part C J. Mech. Eng. Sci..

[13] Mast P.W., Nash G.E., Michopoulos J.G., Thomas R., Badaliance R., Wolock I. (1995). Characterization of strain-induced damage in composites based on the dissipated energy density part I. Basic scheme and formulation. Theor. Appl. Fract. Mec..

[14] Michopoulos J.G., Hermanson J.C., Furukawa T. (2008). Towards the robotic characterization of the constitutive response of composite materials. Compos. Struct..

[15] Michopoulos J.G., Hermanson J.C., Furukawa T., Iliopoulos A. A framework for the automated data-driven constitutive characterization of composites. Proceedings of the 17th International Conference on Composite Materials, ICCM-17.

[16] Michopoulos J.G., Hermanson J.C., Iliopoulos A., Lambrakos S.G., Furukawa T. (2011). Data-driven design optimization for composite material characterization. J. Comput. Inf. Sci. Eng..

[17] Chen B., Zeng Y., Wang H., Li E. (2021). Approximate Bayesian Assisted Inverse Method for Identification of Properties of Variable Stiffness Composite Laminates. Compos. Struct..

[18] Bouhala L., Makradi A., Belouettar S., Younes A., Natarajan S. (2015). An XFEM/CZM based inverse method for identification of composite failure properties. Compos. Struct..

[19] Su M., Peng H., Yuan M., Li S.F. (2021). Identification of the interfacial cohesive law properties of FRP strips externally bonded to concrete using machine learning techniques. Eng. Fract. Mech..

[20] Alfano M., Lubineau G., Paulino G.H. (2015). Global sensitivity analysis in the identification of cohesive models using full-field kinematic data. Int. J. Solids Struct..

[21] (2017). Compressive Residual Strength Properties of Damaged Polymer Matrix Composite Plates.

[22] Morris M.D. (1991). Factorial Sampling Plans for Preliminary Computational Experiments. Technometrics.

[23] Sobol I.M. (1990). On sensitivity estimation for nonlinear mathematical models. Mat. Model..

[24] Mcrae G.J., Tilden J.W., Seinfeld J.H. (1982). Global sensitivity analysis—A computational implementation of the Fourier Amplitude Sensitivity Test (FAST). Comput. Chem. Eng..

[25] Tarantola S., Gatelli D., Mara T.A. (2006). Random balance designs for the estimation of first order global sensitivity indices. Reliab. Eng. Syst. Saf..

[26] Gao B., Yang Q., Peng Z.J., Xie W.H., Jin H., Meng S.H. (2020). A direct random sampling method for the Fourier amplitude sensitivity test of nonuniformly distributed uncertainty inputs and its application in C/C nozzles. Aerosp. Sci. Technol..

[27] Saltelli A., Tarantola S., Chan K.P.S. (1999). A Quantitative Model-Independent Method for Global Sensitivity Analysis of Model Output. Technometrics.

[28] Huang C.H., Wu H.H. (2006). An inverse hyperbolic heat conduction problem in estimating surface heat flux by the conjugate gradient method. J. Appl. Phys..

[29] Tihonov A.N. (1963). On the solution of ill-posed problems and the method of regularization. Dokl. Akad. Nauk SSSR.

[30] Gnanasekaran N., Balaji C. (2011). A Bayesian approach for the simultaneous estimation of surface heat transfer coefficient and thermal conductivity from steady state experiments on fins. Int. J. Heat Mass Transf..

[31] Lee D., Choi D. (2019). Analysis of the Reliability of a Starter-Generator Using a Dynamic Bayesian Network. Reliab. Eng. Syst. Saf..

[32] Li C., Mahadevan S., You L., Choze S., Wang L. (2017). Dynamic Bayesian Network for Aircraft Wing Health Monitoring Digital Twin. AIAA J..

[33] Maybeck P.S. (1990). The Kalman Filter: An Introduction to Concepts. Autonomous Robot Vehicles.

[34] Julier S.J., Uhlmann J.K. (1999). A New Extension of the Kalman Filter to Nonlinear Systems. Proc. SPIE Int. Soc. Opt. Eng..

[35] Fan J., Yung K.C., Pecht M. (2014). Prognostics of Chromaticity State for Phosphor-Converted White Light Emitting Diodes Using an Unscented Kalman Filter Approach. IEEE Trans. Device Mater. Reliab..

[36] Arulampalam M.S., Maskell S., Gordon N., Clapp T. (2002). A tutorial on particle filters for online nonlinear/non-Gaussian Bayesian tracking. IEEE Trans. Signal Process..

[37] Ye Y.M., Yang Q., Yang F., Huo Y.Y., Meng S.H. (2020). Digital Twin for the Structural Health Management of Reusable Spacecraft: A Case Study. Eng. Fract. Mech..

[38] Wang R.G., Zhang L., Zhang J., Liu W.B., He X.D. (2010). Numerical analysis of delamination buckling and growth in slender laminated composite using cohesive element method. Comput. Mater. Sci..

[39] Endalew A.M., Woo K., Kim I.G., Choi D., Kim H.S. (2021). Buckling and delamination growth behavior of composite laminates with circular initial delamination. J. Mech. Sci. Technol..

